# RECONCEPTUALIZING REHABILITATION RESEARCH VIA AN ENACTIVE FRAMEWORK AND A RADICALLY INTERDISCIPLINARY CROSS-ANALYSIS: A STUDY PROTOCOL ON FATIGUE IN POST COVID-19 CONDITION (PCC)

**DOI:** 10.2340/jrm.v57.42254

**Published:** 2025-04-06

**Authors:** Richard LEVI, Ulrika Birberg THORNBERG, Ida BLYSTAD, Anestis DIVANOGLOU, David ENGBLOM, Felipe LEÓN, Sofia Morberg JÄMTERUD, Kristin ZEILER

**Affiliations:** 1 Department of Rehabilitation Medicine and Department of Medical and Health Sciences, Linköping University, Linköping, Sweden; 2 Department of Radiology Linköping, and Department of Health, Medicine and Caring Sciences, Linköping University, Linköping, Sweden; 3 Centre for Medical Image Science and Visualization (CMIV), Linköping University, Linköping, Sweden; 4 Department of Biomedical and Clinical Sciences, Linköping University, Linköping, Sweden; 5 Centre of Philosophy, School of Arts and Humanities, University of Lisbon, Lisbon, Portugal; 6 Department of Thematic Studies: Technology and Social Change and the Centre for Medical Humanities and Bioethics, Linköping University, Linköping, Sweden

**Keywords:** fatigue, interdisciplinary study, models, biopsychosocial, neurological rehabilitation, post-acute COVID-19 syndrome

## Abstract

**Objective:**

To present a radically interdisciplinary research approach to ill-defined symptoms, with a focus on fatigue as a major symptom of post COVID-19 condition, where multiple and, to date, rarely combined approaches may yield a fuller understanding of these symptoms.

**Design:**

Protocol for a mixed-methods study comprising an interdisciplinary cross-analysis.

**Patients:**

35 persons with post COVID-19 condition and severe fatigue were included, and 35 age-, sex-, and educationally matched controls who recovered from COVID-19 without post COVID-19 condition.

**Methods:**

Participants were assessed by a multidisciplinary research team as follows: physician assessment; blood and urinalysis; spirometry and physical performance tests; neuropsychological tests; structural and functional magnetic resonance imaging; extended immunological tests (cytokines); and qualitative phenomenological analysis of interviews. Data will be analysed in accordance with established methods in each of these research fields and by a cross-analysis methodology developed from within an enactive framework. This framework encompasses a focus on neuroscientific, physiological, *and* experiential aspects of the person as a living being in their sociocultural world.

**Conclusion:**

The biopsychosocial model needs to be implemented in research according to methods that allow radically different research paradigms, typically seen as incommensurable, to inform each other in a non-reductionist manner. One application of such an approach is therefore described.

## LAY ABSTRACT

Several of the most debilitating symptoms in medicine, such as fatigue and pain, are highly non-specific and both difficult for the patient to describe and for the caregiver to interpret. The underlying factors contributing to such symptoms are often complex and multiple. Our purpose is to develop a cross-analysis methodology, developed from within an enactive interpretation of the biopsychosocial model that allows for a focus on neuroscientific, physiological, and experiential aspects of the person as a living being in their sociocultural world, and to do so in order to achieve a more holistic and clinically clarifying way of understanding complex conditions, such as post-COVID fatigue. Traditionally conceived, the biopsychosocial model of disease acknowledges this complexity, but does not as such suggest how to capture it for clinical or research purposes. In this paper, we propose a radically interdisciplinary approach, where experts from basic medical science, clinical medicine, psychology, phenomenology, and ethics collaborate, each with their specific perspectives and methods. We then combine and enrich the obtained results through a cross-analysis. We suggest that an enactively modernized biopsychosocial framework is especially suitable for this collaboration, as it acknowledges the close interrelation between mind, body, and environment, and can facilitate cross-analyses of data in this project.

In medicine, it is well known that many patients experience long-term functional impairments and reduced well-being related to poorly understood symptoms. One such example is fatigue, which is present in a wide variety of disorders, including post COVID-19 condition (PCC). Typically, standard clinical assessment only rarely brings clarity as to the specific cause of fatigue and similarly ill-defined and diffuse symptoms, probably due to the diverse mechanisms underlying the genesis and persistence of such symptoms. The biopsychosocial model, first described by Engel (1), was designed to investigate the complex and multifactorial aetiology of such symptoms in a non-reductive manner. Even though this model has been broadly adopted for several decades, at least in theory (see, e.g., Bolton & Gillett [2]), the dominant clinical and research paradigms remain largely sequestered due to their focus on one or the other of the model’s components rather than bringing them to interact with one another. The biopsychosocial model has been criticized for failing to fulfil its promise and failing to account for *how* its different components or dimensions (the “bio”, “psycho”, and “social”) relate to each other (3–5). With de Haan’s (4, p.473) formulation, the biopsychosocial model runs into “the integration problem”, and begs the question “how do body, mind, and world relate?”

This “integration” question has been central in so-called *enactive* approaches since the 1990s. From their inception, enactive approaches have combined insights from phenomenological philosophy, living systems theory in biology, cognitive sciences, and neurosciences (6, 7). This makes an enactive approach particularly suitable for this project, which takes a holistic stance towards the patient *and* combines perspectives and methods from biomedical sciences (rehabilitation medicine and the neurosciences) and the humanities (qualitative phenomenological philosophy). Although the precise relationship between enactive approaches and the biopsychosocial model remains contentious, we follow the recent call for an “enactive modernization” (3, p.2274, see also [8]) of the biopsychosocial model, which appeals to the resources of enactivism in order to fulfil the biopsychosocial model’s promise of shedding light on how biological, psychological, and social factors are integrated with each other.

Despite differences among enactive approaches, they share a broad understanding of cognition as arising from sense-making interactions of an embodied organism with the environment to which it is dynamically coupled (6, 7, 9). They take cognition to be *embodied* (rather than reducible to the internal manipulation of mental representations) and *embedded* in so far as cognitive processes depend on the environment in which the agent is located. Further, enactive approaches agree on the value and need for a “*mutual circulation*” between analyses that focus on “our biological and phenomenological embodiment – i.e., between our existence as living organisms, and our experience of ourselves as lived bodies” (10, p.572; see also [6]).

The aim of the present article is to describe the study protocol of a radically interdisciplinary research study on the symptom of fatigue in the context of PCC. The study is radically interdisciplinary in the sense that it combines perspectives and methods from within both biomedicine and the humanities that, to date, have rarely been combined. Further, it adheres to an enactive framework that acknowledges a foundational unity of the living person as embodied subjectivity *and* as a living organism located in its sociocultural and material environment. The enactive approach is particularly apt, as it invites the combination of third-person perspective assessments and analyses that focus on subjectivity and the patient’s first-person narrated lived experiences. Specifically, by mapping the results of methods focusing on what may be termed as different loci within an enactive approach (see Fig. 1) and then cross-analysing these results, we aim to investigate subjectivity, affectivity, and neural processes of fatigue in PCC fatigue in a more holistic and clinically clarifying way than has typically been the case in the past. Further, we hope that a cross-analysis that brings rehabilitation sciences, neuropsychology, neurobiology, neuroradiology, and qualitative phenomenological philosophy into dialogue with each other may inspire further translational research, creating a dialogue for the sake of our patients. To put this differently, while it can be regarded as conventional to combine perspectives from, for example, neuropsychology and neuroradiology in interdisciplinary medical research, it is much rarer to also include the research strand of qualitative phenomenological philosophy, i.e., a research strand from within the humanities that specifically attends to subjectivity, lived experiences, structures of experience, and modal alterations, as will be further discussed below. Doing so, as in this study protocol, can stimulate the budding discussions on rethinking rehabilitation research.

**Fig. 1:**
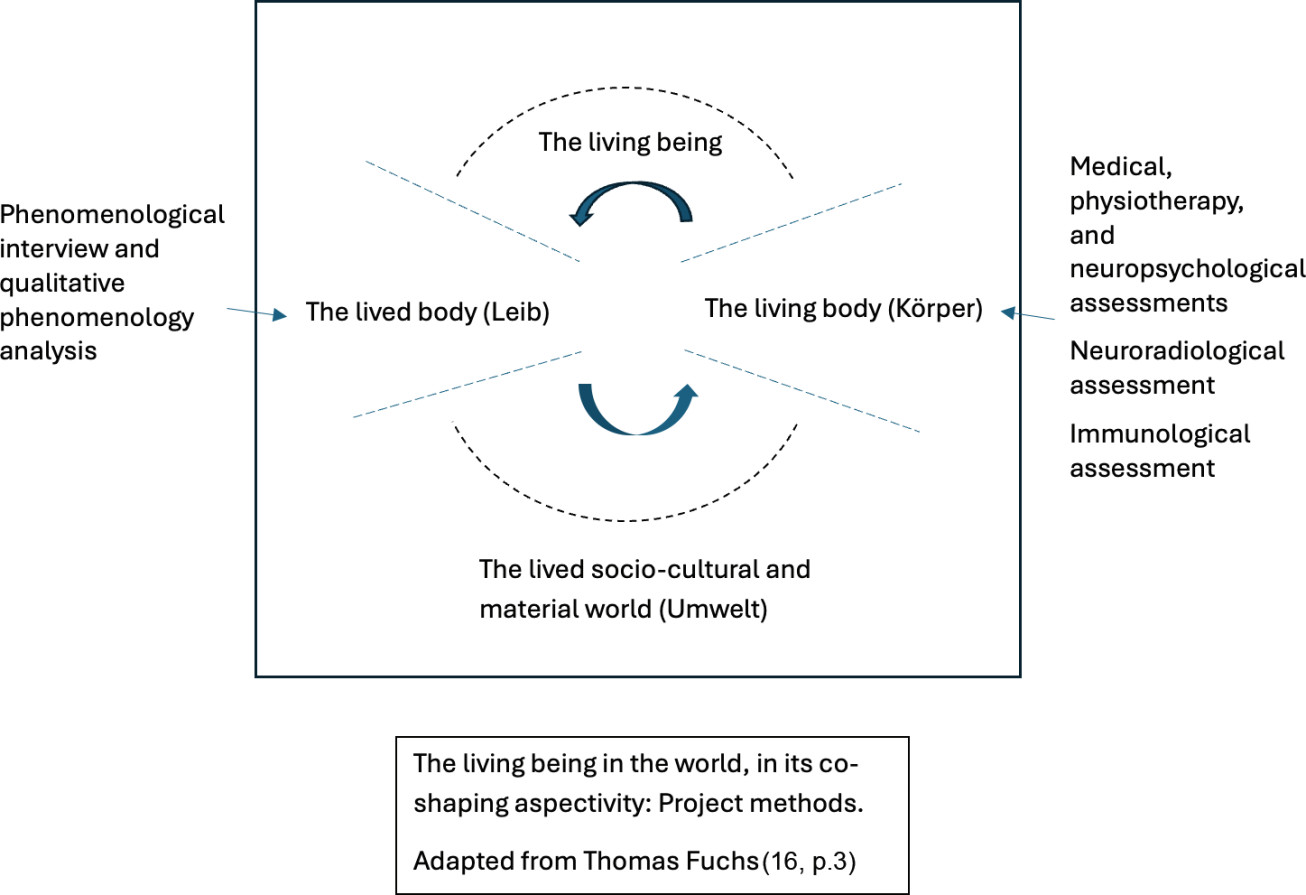
**Visualization of the framework and the methods brought together in the project.** The dotted lines are intended to visualize (*i*) that the lived body is not neatly set apart from the living body, and (*ii*) the embeddedness of the living being, acknowledging the dynamic interplay between the living being and its world. Further, the figure is intended to visualize and acknowledge that while a certain perspective and method may predominantly focus on the production of knowledge concerning the lived body as subjectively lived or the production of knowledge concerning the living body from a third-person perspective, these kinds of knowledges are also brought together and cross-analysed with the overarching aim of gaining a deeper understanding of the experience, characteristics, and pathophysiology of the object of study, that is, fatigue in PCC. The figure is adapted from Fuchs (16, p.3), with his permission. Fuchs' figure (16) is published in Frontiers of Psychology, and Frontiers articles are published under the CC-BY licence, https://creativecommons.org/licenses/by/4.0/

The study’s overarching research questions are:

- How, if at all, do phenomenologically distinct features of the fatigue experiences relate to distinct clinical features?
- Can the analysis of subjectivity and lived experience of fatigue in PCC, in combination with clinical investigations, contribute to conceptual development and clarity, as well as to the development of interdisciplinary analytical methods that combine such methodologies?

The specific research questions are:

- How do patients experience fatigue in PCC? Can phenomenologically different qualities of fatigue in PCC be identified? How do phenomenological structures (such as temporality or affectivity) help constitute the meaning and character of the experiences of fatigue in PCC?
- Do patients with fatigue in PCC differ from a matched control group who have recovered from COVID-19 and are not suffering from PCC as regards the clinical data, the cytokine profile, and tissue properties and connectivity of the brain as investigated with magnetic resonance imaging (MRI)?

## METHODS

### Study design

A mixed-methods study design comprising an interdisciplinary cross-analysis developed from within an enactive framework.

### Setting

The project brings together researchers from clinical settings, the neurosciences (rehabilitation medicine, neuropsychology, neurology, neurobiology, and neuroradiology), and the humanities (philosophy, ethics, scholars trained in combining qualitative research with philosophy). The study involves a partnership between Linköping University Hospital (Department of Rehabilitation Medicine) and Linköping University (Centre for Medical Humanities and Bioethics, Center for Medical Image Science and Visualization, and the Department of Biomedical and Clinical Sciences, and the Department of Thematic Studies) in Sweden.

### Framework

The project’s enactive framework adopts a holistic focus, and it emphasizes the value and need for a “*mutual circulation*” between analyses that centre on phenomenological, neuroscientific, and physiological aspects (10). Varela and colleagues ([6]: lxiv–lxv, 236) expressed this in terms of seeking “to open a space of possibilities in which the circulation between cognitive science and human experience can be fully appreciated”.

Of particular relevance for the present study is Thomas Fuchs’s (11) elaboration of the “circular” relation between the “lived body” and the “living body”. The concept of the lived body, or *Leib,* is an established term in phenomenological philosophy, referring to the subjectively experienced body as disclosed from a first-person perspective, while the concept of *Körper* refers to the body as object – what Fuchs refers to as the “living body” (see also, for example, Husserl [12], Merleau-Ponty [13)], Slatman [14]). In phenomenology, however, this is not a clear-cut distinction: the subjective experience of one’s lived body is not isolated from the way one’s body is seen and conceptualized from a third-person perspective – one’s subjective experience of one’s lived body also involves an experience of oneself *as* encountered by others. The lived body and the living body have been understood as corresponding to two different perspectives, which we commonly shift between and combine in everyday life without reflectively noting that we do so (see, e.g., 13, 15). As argued by Fuchs, the lived body and the living body can be understood as a "*dual aspect*" of the living being (11, p.73), and the living being, in its dual aspectivity and engagement with the world, is understood as the “ontological basis for embodied subjectivity on the one hand” and, at the same time, as “the objective body considered by physiology on the other” (16, p.2). Further, “both experiential and physiological processes, the lived body and the physical [living] body” belong “to a more encompassing system, namely, the system of the living being and its environment, or the person and her world – an ecological system that is in continuous development” (16, p.3).

The focus on the lived *and* the living body serves as a basis for the study protocol’s design. It offers a basic starting point: experiential aspects and neuroscientific aspects refer to the same life process of the living being, though with “a wider or a narrower focus” (16, p. 7), and the relation between the lived and the living body is understood in terms of a “circularity of experiential process and (neuro)physiological structure” that “mutually influence and modify each other” (16, p. 9). This invites attention to the dynamic formation of the lived body and the living body as aspects of the living being, and to an inquiry that moves back and forth between a focus on the lived body and on the living body as aspects of the living being in the world.

### Participants (inclusion and exclusion criteria)

Individuals with a history (at least 6 months prior to recruitment) of COVID-19 infection (verified through laboratory or self-test) between 20 and 65 years of age and able to communicate in Swedish were invited to participate in the study if they met the following criteria:

*Patients.* Individuals with PCC *including* fatigue reported to affect daily life to at least a moderate degree or worse, *and* presenting a Multidimensional Fatigue Inventory (MFI; 17) total score ≥ 53 points (i.e., corresponding to clinically significant fatigue; [18, 19]).

*Controls.* Individuals without PCC, with no or insignificant fatigue, *and* presenting an MFI total score <53 points.

*Exclusion criteria.* Individuals with contraindications to MRI examination and those where fatigue could be explained by comorbid conditions were excluded. Individuals with pre- and/or comorbid conditions unrelated to previous COVID-19 that may affect cognition (such as neuropsychiatric conditions, dementia, depression, etc.) were excluded from both the patient group and the control group.

### Procedures (recruitment, screening, sequencing of data collection components)

Two principal recruitment paths were used to identify patients: (i) individuals previously hospitalized for COVID-19 who had previously participated in the Linköping COVID-19 Study (LinCoS; see [20] for details) and who had expressed interest in being contacted in relation to further studies; and (ii) through information posted at regional PCC clinics. Controls were recruited through social media and through information posted in public areas in regional hospitals and at the university.

Individuals who expressed interest in participating were provided with further information and were asked to sign an informed consent. All individuals who signed informed consent were subsequently screened by a rehabilitation specialist for eligibility for assignment to either the patient or control group. Screening consisted of a structured phone interview (according to a protocol described in [20]), MFI, and exclusion of pre-/comorbid condition as already stated. Controls were selected to ensure matching for age, sex, and educational level.

All consenting individuals who met the inclusion criteria were asked to provide blood and urine samples prior to clinical assessment. Clinical assessment typically started with neuropsychological assessment by a clinical neuropsychologist followed by medical and physiotherapy assessment (by both a physician and a physiotherapist) performed at the outpatient clinic of the rehabilitation department in a single session. Total time for completion of this assessment was typically 3–4 h. Brain MRI was performed on a separate occasion, within a couple of weeks at most, and assessed by a neuroradiologist. Participants with PCC and fatigue participated in phenomenological interviews within a few weeks after MRI with a researcher with extensive interviewing experience.

The temporal ordering of the data collection steps was as follows: (*i*) Clinical assessment (neuropsychologist, including cognitive and affective assessments; physician, including blood and urine sampling, medical record review, medical history, physical examination; physiotherapist, including physical performance testing); (*ii*) Neuroradiology; and finally (*iii*) Phenomenological interviews. All data collection was performed within a period of a few weeks for each participant, and clinical assessment was made during a single clinic visit. By initializing data collection by clinical assessment, the confirmation thereby of inclusion criteria and ruling out of exclusion criteria could be definitive prior to subjecting the participant to the remainder of the assessments. Within clinical assessment, neuropsychological testing was performed first in order to avoid inducing fatigue through the subsequent assessments. By concluding the total data collection with interviews for patient participants, experiences elicited by the previous assessments could also be explored.

### Medical assessment

Each individual met with a physician, who took a standard medical history with a particular focus on COVID-19 and any persisting symptoms attributed to the infection. A general clinical examination, including cardiopulmonary auscultation, blood pressure, and basic neurological assessment, was performed. Routine blood and urine samples were assessed (complete blood count, electrolytes, liver function tests, kidney function tests, thyroid stimulating hormone, C-reactive protein, erythrocyte sedimentation rate, urine dipstick). Results were analysed for pathological findings in accordance with clinical norms and reference intervals. Any significant findings as regards comorbidities and abnormal physical findings or lab results were documented, as were data regarding previous COVID-19 infection and persisting symptoms as reported by the participant. Data were specifically assessed for possible relevance for the experience of fatigue.

### Physiotherapy assessment

The physiotherapy assessment comprises: pulmonary function assessment with modified Medical Research Council (mMRC) scale, spirometry using a portable ultrasonic spirometer (MicroLab, Vyaire Medical, Mettawa, IL, USA), maximal inspiratory (MIP) pressure and expiratory pressure (MEP) (MicroRPM, Vyaire Medical), screening of dysfunctional breathing patterns according to Nijmegen (21), and evaluation of physical activity level according to the Grimby and Frändin 6-level activity scale (22). The reference and lower limit of normal (LLN) values for MIP and MEP were determined according to Evans and Whitelaw (23). Spirometry included vital capacity (VC), forced expiratory volume in 1 s (FEV1), and peak expiratory flow (PEF). The European Respiratory Society GLI calculator was used to determine reference and LLN values for spirometry (https://gli-calculator.ersnet.org/index.html). Testing also included assessment of aerobic capacity and endurance in a 6-minute walk test (reference values and LLN according to [24]); and assessment of lower body strength, endurance, and functional fitness in a 1-minute sit-to-stand test (reference values according to [25]; LLN below 2.5th percentile), both of which were complemented with assessment of fatigue and dyspnoea pre- and post-testing using the Borg CR-10 Scale.

Symptoms and/or signs pertaining to dysautonomia were captured in the medical history as documented by the physician, in clinical somatic status as performed by the physician, by findings and recorded subjective symptoms in conjunction with physiotherapeutic testing, and by first-person narratives obtained through interviews (see below).

### Neuropsychological assessment

A comprehensive neuropsychological test battery was used to assess 4 main cognitive domains. The assessment included multiple tests for each domain: attention, working memory, processing speed, and executive function. Attention was measured with the Ruff 2 & 7 (26) and the computerized Conners Performance Test, 3rd edition (27). Working memory was assessed with the forward and backward digit span composite scores (from the Wechsler Adult Intelligence Scale, WAIS-IV) (28) and learning and memory with the Rey Auditory Verbal Learning Test (29). The Coding Test (from the Wechsler Adult Intelligence Scale, WAIS-IV) (28), and Trail Making Test A (30), were used to assess processing speed. Finally, the Color Word Interference Test (composite scores combining condition 3 and 4 from Delis-Kaplan Executive Function System) (31) and Trail Making Test B (30) were administered to evaluate executive function.

Depression and anxiety were assessed with the Hospital Anxiety and Depression Scale (HADS; [32]) as a brief measure for depression and anxiety levels. Sleep problems were assessed with the Insomnia Severity Index (ISI) (33).

The individual results were transformed to standard scores and *t*-scores for each test, according to the test manuals. Thereafter, *t*-scores and scaled scores were categorized as 1.5 SD above or below the standardized mean. Test results for subtests in each domain (i.e., attention, memory, processing speed, and executive function) may be analysed separately or merged into the domains.

### Neuroradiological assessment

The brains of the participants were examined in a 3T MR scanner (Prisma, Siemens, Erlangen, Germany) using a 64-channel head coil. Acquired anatomical images were: T1-weighted images (T1WI) (repetition time (TR) = 2,300 ms; echo time (TE) = 2.26 ms; flip angle = 8°; matrix = 256 x 256; voxel resolution = 1 x 1 x 1 mm), and T2FLAIR-weighted images (T2FLAIRWI) (TR = 5,000 ms; TE = 386 ms; inversion recovery time (TI) = 1 800 ms; flip angle = 120°; matrix = 256 x 256; voxel resolution = 1 x 1 x 1 mm). Blood oxygen-level-dependent (BOLD) data (resting state functional magnetic resonance imaging: rs-fMRI) was acquired with an echo-planar imaging (EPI) sequence (TR = 761 ms; TE = 24 ms; flip angle = 56°; voxel resolution = 3 x 3 x 3 mm; number of slices collected 45). The collected resting state run lasted 10.16 min. The multi-shell diffusion sequence consisted of 105 diffusion directions (1 volume at b = 0, 4 directions at b = 300 s/mm^2^, 20 directions at b = 1 000 s/mm^2^, 15 directions at b = 2,000 s/mm^2^, 40 directions at b = 3,000 s/mm^2^, 25 directions at b = 4,000 s/mm^2^; TR 3,500 ms; TE 75 ms; voxel resolution 2 x 2 x 4 mm; number of slices: 38). Relaxometry was acquired with Qmap, a multidynamic, multiecho sequence (TR = 4,500 ms; TE1 = 23 ms; TE2 = 106 ms; TI = 25 ms; flip angle = 150°; voxel resolution 0.7 x 0.7 x 4 mm, number of slices = 30).

The morphological T1W and T2FLAIRW images were collected for assessment of any clinically relevant incidental findings. The T1W images were also used as a template for registration and mapping in the analysis of the quantitative sequences of BOLD, diffusion, and the Qmap.

In terms of the connectivity of the brain, the rs-fMRI provides important information and has been used in several studies on PCC, with divergent findings (34, 35). Advanced diffusion provides insight into the microstructural properties of the brain tissue, whereas previous research indicates changes of the white matter in PCC (36). Relaxometry is a method that measures the quantitative relaxation values of the tissue, which enables objective assessment of brain properties such as volume and myelin content (37). This can enable detection of brain changes that are not visible on conventional morphological MR images.

The initial analysis will be performed on a global whole-brain basis comparing the 2 groups. The rs-fMRI analyses will be guided by the cross-analysis discussions and findings from the other steps of the study in a hypothesis-driven manner.

### Immunological assessment

Following venous blood sampling, BD Vacutainer EDTA tubes were centrifuged at 2,500 g for 10 min. Plasma was transferred to a new tube and kept refrigerated during transport to the storage facility. There, samples were aliquoted and stored at –80°C. Plasma samples were thawed, and samples were transferred to 96 well plates for further analysis using a customised Luminex™ multiplex assay according to the instructions of the R&D Systems, Abingdon, UK. Briefly, 50 μL of diluted sample and 50 μL of magnetic, premixed, microparticle cocktail with antibodies specific for each mediator were mixed in each well and incubated in an orbital shaker. Samples were washed using a magnetic bead separator (HydroFlex™, Shrewsbury, UK) and incubated with a premixed biotin-antibody cocktail with antibodies specific for each cytokine/chemokine. Antibody/analyte complexes were stained with Streptavidin-PE, and plates were read using a Luminex FLEXMAP 3D w/xPONENT 4.3 instrument. A standard curve was generated for each analyte to convert median fluorescence intensity to concentrations (typically pg/ml). Molecular targets were selected based on previous studies (38–47) and included ADAMTS13, APP, CCL4, CCL11, CCL19, CXCL5, Galectin-1, IFN-beta, IL-1 beta, IL-1 RII, IL-6, IL-7, IL-8, MMP-1, MMP-8, P-Selectin, TNF-alpha, and vWF-A2.

### Phenomenological interviews and qualitative phenomenological analysis

Phenomenological semi-structured interviews were conducted, focused on the lived experience of fatigue in PCC. Thus, interviews were performed only with participants with PCC and fatigue. The interviews were designed to invite the interviewees to narrate their experience of fatigue, their experience of falling ill and being ill with fatigue in PCC, what this meant and felt like for them, and how they experienced themselves, their bodies, and their engagement with others and their environment in the context of illness. The interview guide was “front-loaded” in the sense that basic phenomenological foci were included in the interview guide (such as embodiment) (48, 49).

Phenomenological philosophy investigates subjectivity and lived experience, with a primary focus on various more-or-less invariant structures (such as embodiment, sociality, and affectivity) that make possible and help shape lived experiences (see, e.g., [13, 15]). It also focuses on socioculturally contingent yet constitutive structures (such as prevailing understandings of some illnesses as contested) that help shape lived experiences (50). Against this background, phenomenological philosophy allows for an investigation of overall alterations of modes of being in the world. Qualitative phenomenological philosophy, here referred to as “qualitative phenomenology”, investigates and offers accounts of the interviewees’ narrated lived experiences by identifying and analysing specific alterations in modes of being in the world and other structures of experience, as well as how one’s sense of self, embodiment, others, things, and the world manifest in experience (see [49, 51]). The qualitative phenomenology analysis proceeded in steps that included the following: an initial thematic analysis (coding, identification of subthemes and themes, for an introduction on thematic analysis, see [52]); engagement with phenomenological philosophy in relation to a specific identified theme; re-reading, re-coding, and re-grouping of codes and subthemes into phenomenologically oriented themes with attention to phenomenological structures and modal alterations; a dialogical process across analysed data and phenomenological literature; conceptual discussion and elaboration to capture nuances within the analysed material (51).

### Sample size

The sample size was established in order to, on the one hand, be deemed minimally large enough to be sufficient to gain statistical power for the quantitative analyses (e.g., neuropsychology and MRI) and, on the other hand, maximally large for feasibility in the qualitative phenomenology analysis.

### The interdisciplinary cross-analysis methodology

The enactive approach provides a framework for the combination of different perspectives and methods within this study protocol. Some of these focus predominantly on the living body, i.e., the objective body, whereas others focus predominantly on the lived body, i.e., on embodied subjectivity. Centrally, the lived body and the living body are not understood as neatly set apart, but as intertwined with each other (see Fig. 1). More specifically, the perspective that predominantly focuses on the body as subjectively lived still also attends to the living body; perspectives that predominantly focus on the living body can still gain from being cross-read with insights from perspectives that predominantly attend to the lived body, and processes within both the lived body and the living body can mutually shape each other.

As seen, the enactive approach allows for attention to a “non-linear relation” (53) between different dimensions (in our case, the phenomenal, the neuropsychological, and the physiological/neuroscientific). More precisely, it allows us to assemble and analyse aspects that may contribute to the phenomena of fatigue in PCC in a non-reductive and non-fusional way, while still allowing room for distinctions and differentiations, not only among different contributing factors (for a discussion of the problem with the idea of fusion in this context, see Gallagher [54]), but also among different kinds of knowledge production practices. The enactive approach allows us to move in between different loci of analysis and to understand the lived body and the living body as belonging to an encompassing and dynamic system of the person in their world, and this is central for the project’s cross-analysis.

The cross-analysis methodology that we develop rests on the project’s framework. It entails several steps. First, each research participant participates in all assessments and interviews (except the control group, which is not part of the phenomenological interview), and assessments and interviews are analysed through the distinct methods used in each discipline/research field. In the next step, all results, from all the perspectives and methods above, are put in a shared Excel sheet (Microsoft Corp, Redmond, WA, USA) to allow for additional analysis across these. The different team members present the result of the specific analyses, with the aim of allowing the team as a whole to move between different results from the different analyses, discussing them in relation to each other, investigating whether or not the different results resonate with each other and, in any case, how the resonance or lack thereof can be understood and further contribute to the understanding of fatigue in PCC. This design is intended to allow us, as a team, to zoom in and out and attend to, for example, “local” neurobiological processes and more “global” alterations in patients’ experiences of their relation to the world, which we then discuss in relation to each other (cf. Coninx and Stilwell [53]).

The cross-analysis entails an inquiry *across* the results from the distinct analyses, as outlined earlier, and it may commence from the results of any of the steps and proceed with discussions of these results from that step in relation to results in the other steps. Further, based on this cross-discussion of the results, further analytic steps can follow. Concretely, the results from the qualitative phenomenological analysis, for example in terms of possible identified affective modes, can be used to offer guidance for further regional analyses in the neuroradiology analysis in a hypothesis-driven manner, to explore possible connections or lack of these. As another example, results from the neuropsychological assessment may be used to open up for cross-discussions concerning cognitive-affective dimensions identified in the neuroradiological and the qualitative phenomenological analyses, and again, for further hypothesis-driven analysis.

From a process perspective, as team members are experts in the specific analysis they perform, they present their distinct analysis at team meetings, and the team as a whole engages in conversations about connections between, and differences across, the distinct results from within the analyses. These conversations are central throughout the project as a whole, from its very inception, to create a basic shared understanding of what the different perspectives and methods contribute, as well as their limitations, and they are crucial to the cross-analysis itself.

### Informed consent and ethics

The Swedish Ethical Review Authority approved the study (Dnr 2022-02221-01/ 2022-03823-02) prior to recruitment. All research activities were conducted according to the Helsinki Declaration. Participation in the project was voluntary based on informed consent, and the participants could withdraw at any time without giving a reason. Not participating or withdrawing had no implications for the provision of healthcare services.

## DISCUSSION

The study protocol presented in this paper has two main novelties. First, it suggests that a qualitative phenomenological analysis of the interviews can contribute to more conventional interdisciplinary medical research, and bring insights on subjectivity, lived experiences, structures of experience and modal alterations concerning, in this case, experiences of fatigue in PCC. What the qualitative phenomenological analysis specifically can be expected to contribute is not only a focus on first-person narrated experiences, as is the case in different ways for the whole of the field of qualitative research, but an analysis of, for example, how fatigue in PCC can alter patients’ very ways of perceiving and experiencing themselves, others, space, embodiment and their world. As another example, such an analysis is apt to identify and examine different affective modes when living with fatigue in PCC, being attentive to how these help shape the way selves experience their surrounding world. Such insights, from within this kind of analysis, should matter to rehabilitation medicine, in the sense that they contribute to the understanding of the experiential dimension of a particular illness, which is arguably of pivotal importance in the clinical encounter.

Second, however, our specific way of combining in the cross-analysis perspectives and methods from these distinct research strands, via an enactive approach, is also, to our understanding, novel. While enactive frameworks are far from new, much enactive literature that offers analyses that engage with empirical results attentive to the lived body and the living body do so by discussing such results from *others’* research or contributing an analysis from primarily one of the loci above (see Fig. 1). By contrast, this project engages with data and results that are generated within the project itself, for the sake of deepening the understanding of fatigue in PCC, while being attentive to the lived body and the living body as aspects of the living being in the world, in a systematic, non-reductive, and non-fusional way. To our knowledge, the project’s cross-analysis is a new method, though it builds on and is developed from within enactive approaches. It is performed by researchers with saliently different academic backgrounds who work closely together. Further, we believe that, to implement the biopsychosocial model in all its complexity, concrete steps must be taken to bring together and combine widely diverging research paradigms. This requires an openness towards a non-reductionist dialogue for the sake of fruitfully bringing together and cross-analysing relevant data.

### Challenges related to radically interdisciplinary research

Working across disciplines from the natural sciences and biomedicine, on the one hand, and the humanities, on the other hand, is admittedly demanding. Research paradigms, vocabularies, deep-seated prior assumptions, and standards for presenting research in scientific journals vary considerably between these epistemic fields. Thus, an openness and non-dogmatic attitude towards unfamiliar perspectives are essential. A non-reductionistic approach is also called for, where the different perspectives may inform one another in order to shed light on the topic under study, rather than imposing non-commensurable research paradigms on each other. To achieve the aim of developing such an approach, a willingness to engage with colleagues from very different disciplines, to listen to, and seek to reach a basic understanding of unfamiliar methods and perspectives is required, while also respecting different areas of expertise and competences within the research team.

### Significance and limitations

A definite significance of adopting the radically interdisciplinary approach described in this protocol is the implementation of a methodology that takes seriously the enactively modernized biopsychosocial paradigm, by allowing a more holistic and nuanced understanding of the life-world of the patient, and thereby potentially clarifying where and how interventions are called for. This is also in alignment with the intentions of the International Classification of Functioning, Disability and Health (ICF) taxonomy, which includes assessments pertaining to the biopsychosocial paradigm, for example, bodily structure and function, activity and participation, and environmental factors. Limitations that perhaps are inevitable, although manageable, include the compromises that are needed to balance the requirements of large sample sizes in quantitative research with the maximal limits in terms of sample size in qualitative research. That being said, we believe that this framework can prove fruitful for research aiming to implement and fulfil the promise of the biopsychosocial paradigm, especially in light of the holistic ethos that is inherent to rehabilitation research.
